# GenePiper, a Graphical User Interface Tool for Microbiome Sequence Data Mining

**DOI:** 10.1128/MRA.01195-19

**Published:** 2020-01-02

**Authors:** W. M. Tong, Yuki Chan

**Affiliations:** a Faculty of Dentistry, The University of Hong Kong, Pokfulam, Hong Kong SAR; Indiana University, Bloomington

## Abstract

Amplicon sequencing of the 16S rRNA gene is commonly performed for the assessment and comparison of microbiomes. Here, we introduce GenePiper, an open-source R Shiny application that provides an easy-to-use interface, a wide range of analytical methods, and optimized graphical outputs for offline microbiome data analyses.

## ANNOUNCEMENT

The profiling of microbiomes by high-throughput amplicon sequencing has become a standard approach in many research disciplines. Many bioinformatics tools have been developed to analyze such data, in standalone or online applications (1–5). However, most of these tools are command line based and have a steep learning curve for general users. Many of the packages require numerous sources of dependencies with different compatibilities, which complicates their installation and maintenance, whereas Web applications have data transfer and computation time constraints, especially for huge data sets. The uploading of data onto online servers also raises data security concerns. To address these issues, we developed GenePiper, an open-source R Shiny application, based on a virtual environment, that provides a united offline system for microbiome data analyses.

GenePiper is an open-source R Shiny application built in a virtual Linux environment. It depends on VirtualBox (Oracle) and Vagrant (HashiCorp, USA), which are available on Windows, Mac-OS X, and Linux platforms. Users download the GenePiper Vagrant configuration file, which Vagrant uses to set up the R Shiny (6) server, with all of the applications, within a virtual environment on the local computer. The main interface of GenePiper is accessed locally through a Web browser (such as Chrome, Firefox, or Safari).

**FIG 1:**
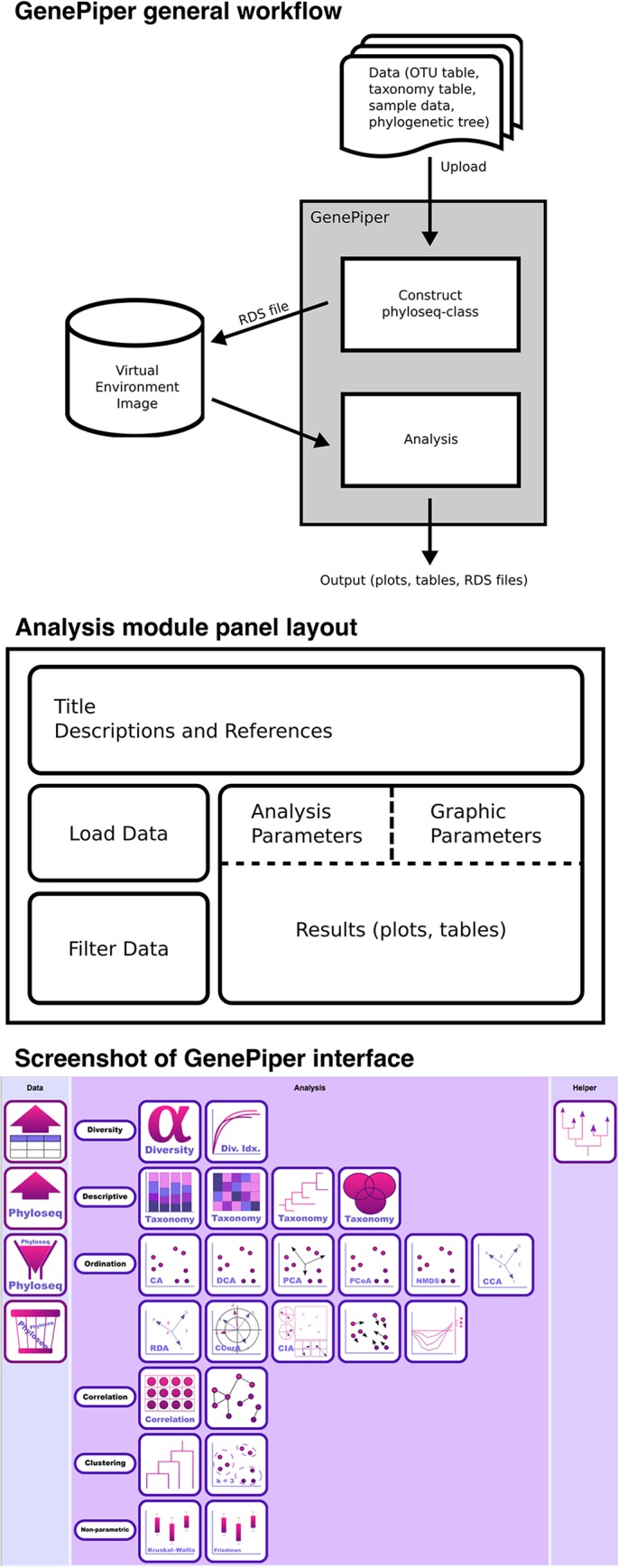
(Top) GenePiper general workflow. Data loaded into GenePiper are formatted into a phyloseq-class structure and stored in the virtual environment as an RDS file. Downstream analytical modules recall this RDS file for analysis. (Middle) Analysis module panel layout. All of the analytical modules in GenePiper share the same layout, with a top panel that shows the title, description, and references, a bottom-left panel for loading and filtering data, and a bottom-right panel for setting up the analysis parameters and displaying the results. (Bottom) Screenshot of the GenePiper interface.

GenePiper requires three input files, namely, an operational taxonomic unit (OTU) table, a taxonomy table, and a sample data table (Fig. 1). It is optional to provide a phylogenetic tree for UniFrac distance calculations (7). These files are loaded into GenePiper via the data import module. GenePiper constructs a “phyloseq-class” data structure with the loaded data and stores it in RDS format in the virtual environment. These RDS data are saved and can be recalled by a unique data label in subsequent analytical modules. Alternatively, a phyloseq-class data object stored in RDS format can be imported into GenePiper for analysis.

GenePiper complements existing packages, with easy access to many popular and well-documented analytical methods, including phyloseq (3), vegan (8), phangorn (9), ape (10), VennDiagram (11), Hmisc (12), SpiecEasi (13), SparCC (14), and many others. The analytical modules are categorized into six broad groups, i.e., diversity analysis, descriptive analysis, ordination, clustering, correlation analysis, and nonparametric statistical tests. GenePiper generates figures mainly using the ggplot R package (15) and provides full control of the graphical parameters. Users may explore their microbiome data with options for visualization including a diversity index curve, taxonomic bar chart and heatmap, phylogenetic tree, Venn diagram, scatterplot ordination such as correspondence analysis, detrended correspondence analysis, principal component analysis, principal coordinate analysis, nonmetric multidimensional scaling, canonical correspondence analysis, redundancy analysis, canonical correlation analysis, coinertia analysis, Procrustes analysis, principal response curve, correlation plot, correlation network plot, clustering dendrogram, and boxplot with nonparametric test.

In summary, GenePiper is an integrated data-mining application in which the routine analytical pipeline can be operated easily using a graphical user interface. GenePiper allows researchers to efficiently test-run different parameter combinations for optimization and for generation of results for publication.

### Data availability.

GenePiper is available at https://github.com/raytonghk/GenePiper. A step-by-step overview tutorial is available at https://github.com/raytonghk/genepiper/wiki/01.-Introduction.
